# Comparative Performance of Body Mass Index and Simple Anthropometric Indices for Identifying Body Fat Percentage-Defined Obesity

**DOI:** 10.3390/healthcare14121688

**Published:** 2026-06-12

**Authors:** Hend A. Algrvi, Madhawi M. Aldhwayan, Sara Al-Musharaf, Tagreed A. Mazi, Afshan Masood, Hicham Benabdelkamel, Ghadeer S. Aljuraiban

**Affiliations:** 1 Department of Community Health Sciences, College of Applied Medical Sciences, King Saud University, Riyadh 11451, Saudi Arabia; algrvi2010@gmail.com (H.A.A.); salmosharruf@ksu.edu.sa (S.A.-M.); tmazi@ksu.edu.sa (T.A.M.); galjuraiban@ksu.edu.sa (G.S.A.); 2 Clinical Nutrition Department, Najran Armed Forces Hospital, Najran 66252, Saudi Arabia; 3 Centre of Excellence in Biotechnology Research (CEBR), King Saud University, Riyadh 11451, Saudi Arabia; 4 Proteomics Resource Unit, Obesity Research Centre, College of Medicine, King Saud University, Riyadh 11451, Saudi Arabia; afsmasood@ksu.edu.sa (A.M.); helkamel@ksu.edu.sa (H.B.)

**Keywords:** obesity, anthropometric indices, waist circumference, body mass index, body fat percentage

## Abstract

**Background/Objectives**: Obesity is a major public health concern, and accurate diagnosis is essential for effective treatment and prevention strategies. Obesity screening utilizes anthropometric indices, among which body mass index (BMI) has been most widely used; however the comparative performance of alternative indices still needs to be evaluated. **Methods**: This cross-sectional study included 1042 participants. Anthropometric indices including BMI, waist circumference (WC), waist-to-height ratio (WHtR), waist–hip circumference ratio (WHR), neck circumference (NC), and hip circumference (HC) were measured among Saudi adults. Body fat percentage was measured using bioelectrical impedance analysis and used to define obesity. Correlation analysis was used to assess associations with BF%, and receiver operating characteristic curve analysis was used to compare discriminatory performance and determine optimal obesity-screening cutoffs using the Youden index. **Results**: The study included 1042 Saudi adults, predominantly women (61.9%), with a mean age of 31.8 ± 12 years. BMI showed the strongest overall correlation with body fat percentage (r = 0.707), followed by hip circumference and waist-to-height ratio. ROC curve analysis revealed excellent discriminatory performance for BMI in the total cohort (AUC = 0.924, 95% CI: 0.908–0.939), followed by HC (AUC = 0.887) and WHtR (AUC = 0.861); the least discriminatory performance was for WHR (AUC = 0.667). Further sex-stratified analysis showed that BMI had the strongest correlation with BF%, in both men (r = 0.837), and women (r = 0.821). On the other hand, ROC analysis showed that WHtR was the best in men (AUC = 0.932), with performance comparable to BMI, whereas BMI remained the strongest discriminator in women (AUC = 0.930). **Conclusions:** BMI demonstrated the highest discriminatory performance for identifying BF-defined obesity in this cohort, supporting its value as a practical and reliable screening index. Although waist circumference and waist-to-height ratio reflected differences in body fat distribution, they did not outperform BMI. These findings support the continued use of BMI as the primary obesity screening measure, while highlighting the importance of using sex-specific interpretation of complementary anthropometric indices.

## 1. Introduction

Obesity is recognized as a chronic and relapsing disease resulting from multifactorial interactions encompassing genetic predisposition, neurobiological mechanisms, dietary behaviors, food environment accessibility, socioeconomic influences, and broader environmental factors [1]. As of 2022, approximately 16% of adults aged 18 and older were living with obesity [1]. According to the Saudi Arabian General Authority for Statistics in its 2024 Health Determinants Statistics Publication, 23.1% of individuals aged 15 and older are living with obesity, and 45.1% are overweight [2]. Obesity increases the risk of cardiometabolic diseases such as dyslipidemia, hypertension, and type 2 diabetes, with visceral adiposity, chronic low-grade inflammation, and insulin resistance serving as key underlying mechanisms [3].

Obesity must be diagnosed accurately and promptly for effective intervention. Body mass index (BMI) is widely used as a diagnostic index because of its simplicity. However, it has notable limitations: BMI does not distinguish between lean and fat mass or account for body fat distribution; thus, its use alone can potentially lead to misclassification [4]. Moreover, it is also known that BMI is influenced by age, gender, and ethnicity [5]. To overcome these limitations, central obesity indices such as waist-to–height ratio (WHtR), waist circumference (WC), and neck circumference (NC) have been previously used for assessing obesity [4,5,6]. Although these indices have been studied more in relation to diabetes or cardiometabolic disease and metabolic syndrome, they are also known to be influenced by weight gain and reflect a different aspect of body composition [6]. Specifically, WC and WHtR primarily reflect central adiposity, WHR characterizes fat distribution between the abdomen and lower body, and NC has been linked to upper-body subcutaneous fat and metabolic risk, while HC reflects gluteofemoral adiposity. Moreover, objectively measured body fat percentage (BF%), derived from bioelectrical impedance analysis (BIA) or dual-energy X-ray absorptiometry (DXA), has been used to provide a direct measure of adiposity and is increasingly recognized as a more biologically meaningful measure that enables a more precise distinction between fat and lean mass than does BMI [4]. Body fat percentage was selected as the reference standard for obesity classification in this study. Although BMI is widely used in clinical and epidemiological settings, it is unable to discriminate between fat and lean mass, and its diagnostic accuracy for excess adiposity is limited, particularly in the intermediate BMI range [7]. Body fat percentage was assessed using bioelectrical impedance analysis (BIA), a widely used method in epidemiological research owing to its non-invasive nature, portability, and cost-effectiveness [8]. However, measurements are susceptible to variations in hydration status, recent food intake, and physical activity level, which may introduce measurement error [9]. While dual-energy X-ray absorptiometry (DXA) is considered the reference standard for body composition assessment due to its precision and ability to differentiate regional fat and lean mass, its limited accessibility, radiation exposure, and higher cost restrict its use in large-scale epidemiological studies [8]. BIA has nonetheless demonstrated acceptable agreement with DXA in population-based settings, supporting its use as a practical alternative when DXA is not feasible [10]. According to a systematic review and meta-analysis, BMI has acceptable specificity for identifying excess adiposity at the individual level, but as its sensitivity is poor, it does not classify individuals with excessive BF% as obese [5]. Excess BF% is known to be linked to metabolic dysregulation independent of body weight; therefore, relying solely on BMI for obesity assessment in clinical settings is not advised [5].

In epidemiological research and clinical practice across various nations, ethnic groups and sexes, criteria for defining obesity differ, and diverse methods, including single or multiple indices for diagnosis, are used [11,12,13,14]. In Saudi Arabia, the proposed BMI cutoff for obesity is ≥27 kg/m^2^ [15], which is lower than the global standard of ≥30 kg/m^2^ [16]. Moreover, NC has strong correlations with BMI, WC and cardiometabolic risk factors, with proposed cutoffs of ≥37.5 cm for men and ≥32.5 cm for women [17]. WHtR has exhibited excellent diagnostic potential for assessing central obesity among Saudis; a threshold of ≥0.50 significantly elevates the risks of metabolic syndrome and other cardiovascular risk factors [18].

In previous studies, measures have often been evaluated individually, or the focus has been solely on one or two indices [15,17,18]. To enhance the accuracy of obesity diagnosis, body composition prediction methods must be improved, context-specific cut-off points established, or a combination of indices used [19]. The measures just described, which are accessible and reliable, especially in resource-limited settings, can bridge the gap between feasibility and accuracy, thereby enabling more reliable obesity assessment in real-world settings where advanced techniques may not be practical. In this study, we aim to evaluate and compare the discriminative performance of selected anthropometric indices, i.e., BMI, WC, HC, WHR, WHtR, and NC, in assessing obesity in Saudi adults and determine the optimal cutoff values for each index. The findings are intended to inform evidence-based recommendations on the most suitable indices for use in epidemiological and clinical research within the Saudi population.

## 2. Materials and Methods

This cross-sectional study was conducted at the University Clinics of King Saud University in Riyadh, Saudi Arabia, between February 2023 and January 2024. The study received approval from the local institutional ethics committee (No. 22/0193/IRB). Written consent to participate was obtained from all participants, who were informed of their right to withdraw from the study at any time.

### 2.1. Participants and Recruitment

The current study is part of a large cohort study, the characteristics and recruitment process of which have been reported elsewhere [20]. All participants meeting the inclusion criteria were included, and the available sample size provided substantial statistical power for the primary diagnostic accuracy comparisons. The study included adults aged 18 years and older. Individuals younger than 18, those with conditions that could impair dietary intake, those with chronic illnesses such as renal and hepatic failure and those with a history of malabsorption disorders were excluded. An advertisement detailing the research and its inclusion requirements was disseminated online via social media to notify potential volunteers. To mitigate potential sources of bias, a standardized recruitment strategy was employed, and all participant interactions followed a predefined protocol to ensure uniformity. The research team at the University’s Clinics explained the study aims and addressed any inquiries during the visit.

### 2.2. Data Collection and Tools

Participants were asked to come in the morning after a standard 10 h fast. Participants answered structured questionnaires on sociodemographic information and medical history, and their anthropometric data were collected by trained dietitians using standardized methods. All anthropometric indices were measured twice, and the averages were recorded. Participants’ weight (in kilograms) and height (in centimeters) were converted into meters for BMI calculations, measured in light clothing and without shoes, and recorded to the nearest 0.1 kg and 0.5 cm, respectively. WC, HC, and NC were measured to the nearest 0.5 cm with non-stretchable tape. WC was measured at the narrowest point between the lowest rib and the umbilicus [21]. WC ≥ 102 cm in men and ≥88 cm in women were considered abnormally large [22]. HC was measured at the widest circumference of the buttocks at the level of the greater trochanter, with the legs positioned close together. NC was assessed using a tape measure positioned at the midpoint of the neck, between the chin and collarbone, and BMI was calculated by dividing the weight (in kilograms) by the square of the height (in meters) [21,23]. Participants were categorized as underweight (BMI < 18.5 kg/m^2^), normal weight (BMI 18.5 to <25 kg/m^2^), or overweight (BMI 25 to <30 kg/m^2^) and with obesity (BMI ≥ 30 kg/m^2^) [1]. WHtR and WHR were calculated as the ratios of WC to height and to HC, respectively [21,23]. We analyzed body composition using a calibrated bioelectrical impedance analyzer (770 Bioelectrical Impedance Analyzer; In Body, Seoul, Republic of Korea), which measured BF% and body weight in kilograms. BIA measurements were performed by trained dietitians, all of whom received standardized training from the same experienced investigator prior to data collection. Additionally, the use of a standardized automated BIA device inherently reduces operator-dependent error, as measurements are generated by a fixed algorithm rather than by the operator. To ensure measurement consistency, each participant underwent two consecutive BIA measurements, and the mean value was used for analysis. These procedural steps were implemented to minimize inter-observer variability. Sociodemographic data collected included sex, age, academic level, income, occupation, and marital status.

### 2.3. Statistical Data Analysis

Data for continuous variables were calculated as means and standard deviations, and data for categorical variables were calculated as frequencies and proportions. Multiple-response dichotomy analysis was performed for variables with multiple response options (e.g., “tick-all-that-apply” questions), such as medical history. Histograms and the Kolmogorov–Smirnov test of normality were used to assess the normality of metric variables, and variance inflation factors and tolerance indices were used to assess collinearity or multicollinearity. The strength of the linear association between each anthropometric obesity index was quantified using the Pearson correlation coefficient (r). Correlations were estimated in the overall cohort and based on gender.

In the primary analysis, the discriminative ability of each anthropometric index in identifying obesity, defined by body fat percentage (BF%), was evaluated using receiver operating characteristic (ROC) curve analysis. Following established sex-specific cutoffs, obesity was defined as BF% ≥25% in men and ≥35% in women. The area under the ROC curve (AUC) with 95% confidence intervals was used to assess the sensitivity and specificity of each anthropometric measure. The Youden index (calculated as sensitivity + specificity − 1) was used to determine the optimal cutoff values for each anthropometric index yielding the highest combined sensitivity and specificity. Cutoff points associated with the highest Youden index values were considered preferable as diagnostic thresholds. Given the well-established sexual dimorphism in body composition, whereby men and women differ substantially in fat distribution, fat mass proportion, and adipose tissue physiology, all analyses were performed for the total cohort and stratified by sex. This approach was adopted to ensure that sex-specific differences in the diagnostic performance of anthropometric indices were appropriately captured. Independent-groups nonparametric Z tests were performed to evaluate the statistical significance of differences in AUC values between anthropometric indices within each sex. A significance level of alpha = 0.05 was used in all statistical tests. For all data analysis, we used SPSS Statistics for Windows, Version 28 (IBM Corp., Armonk, NY, USA).

## 3. Results

### 3.1. Participant Characteristics

A total of 1042 participants enrolled in and completed the study. The characteristics of the participants are listed in Table 1. Of the participants, 38.1% were male and 61.9% female; their average age was 31.8 ± 12 years. Approximately one third (35.9%) were married, and >50% had university degrees.

### 3.2. Anthropometric Measurements

Table 2 lists the participants’ anthropometric measures. The mean weight was 72.5 ± 18.9 kg, and the mean BMI was 26.8 ± 6.1 kg/m^2^. Of the participants, 13% were classified as underweight, 30.6% as overweight and approximately 28% as with obesity.

### 3.3. Correlations Between Anthropometric Indices and Body Fat Percentage

Pearson correlations between the six anthropometric indices and BF% are presented in Table 3. In the overall cohort, BMI showed the strongest linear association with body fat percentage (r = 0.707), followed by HC (r = 0.636) and WHtR (r = 0.510). Pooled correlations for WC, NC, and WHR were 0.369, 0.084, and 0.075, respectively.

Gender-based analysis showed that in men, BMI exhibited the strongest correlation with BF% (r = 0.837), closely paralleled by WHtR (r = 0.828) and WC (r = 0.789). HC also showed a strong correlation (r = 0.766), while WHR (r = 0.523) and NC (r = 0.531) were comparatively weaker. On the other hand, in women, BMI again ranked highest (r = 0.821), followed by HC (r = 0.740). WC and WHtR demonstrated equivalent moderate-to-strong correlations (r = 0.687 each), and NC produced a moderate correlation (r = 0.544). WHR showed the weakest association in women (r = 0.304). All correlations were statistically significant (*p* < 0.01 except where noted in Table 3).

### 3.4. Discriminative Performance of Anthropometric Indices for Identifying Obesity

**Figure 1:**
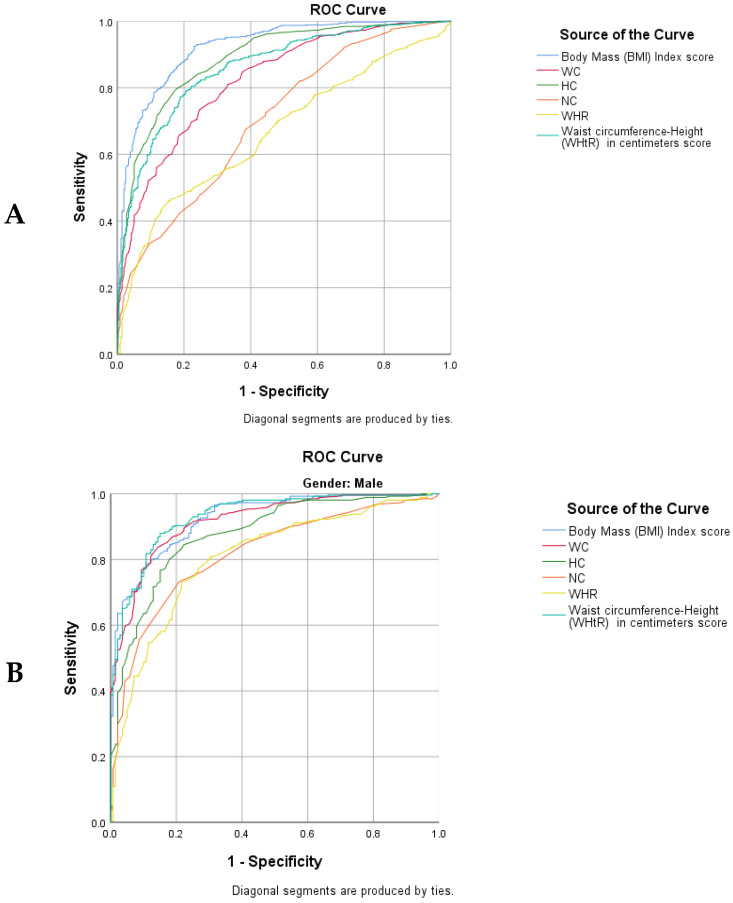

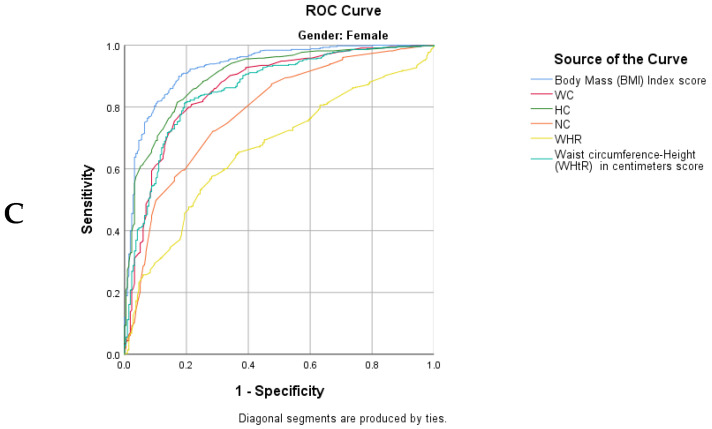
Receiver operating characteristic curves for anthropometric indices in identifying obesity. (**A**) Overall, (**B**) males and (**C**) females. HC, hip circumference; WC, waist circumference; WHR, waist–hip circumference ratio.

The AUC results from the analysis of the discriminative performance of selected anthropometric indices in identifying obesity are presented in Table 4. ROC analysis demonstrated excellent discriminatory performance for several anthropometric indices in identifying obesity defined by BF% (Table 4). In the overall cohort, BMI achieved the highest AUC (0.924, 95% CI 0.908–0.939), corresponding to an optimal cutoff of 23.25 kg/m^2^ with a sensitivity of 92.4% and specificity of 77.0%. HC ranked second (AUC = 0.887; cutoff 101.5 cm), followed by WHtR (AUC = 0.861; cutoff 0.48), and produced the most balanced combination of sensitivity and specificity (HC: 79.6% and 82.3%; WHtR: 79.2% and 79.2%), each approaching 80% on both measures. Waist circumference (AUC = 0.824; cutoff 80.25 cm) achieved comparable sensitivity (73.5%) and specificity (75.3%) at a cutoff of 80.25 cm while NC and WHR displayed substantially weaker discriminatory ability (NC sensitivity 67.5%, specificity 61.5%; WHR sensitivity 46.1%, specificity 84.6%), with WHR capturing fewer than half of the obese participants, Figure 1A.

In the sex-stratified analyses, two distinct diagnostic patterns were revealed. The differences between male and female participants in the AUCs for various obesity indices are listed in Table 5. Among men, three indices, WHtR, BMI and WC, performed comparably well, indicating that any of the three may serve as a primary screening index in this group. WHtR was the strongest discriminator (AUC = 0.932, 95% CI 0.908–0.956; cutoff 0.52) and showed the highest sensitivity (88.0%), as well as high specificity (84.9%); BMI (AUC = 0.925; cutoff 26.32 kg/m^2^) that had the highest specificity (89.2%) of any index with a sensitivity of 78.7%. The third index noted among men with high discriminatory performance was WC (AUC = 0.918; cutoff 92.0 cm), and this index had a high sensitivity (83.7%) and specificity (85.6%). The remaining three indices, HC, NC, and WHR, on the other hand, showed lower but acceptable performance, with sensitivities of 73.3–80.2% and specificities of 78.4–82.0%, Figure 1B.

Among women, BMI emerged as the single strongest discriminator (AUC = 0.930, 95% CI 0.911–0.949; cutoff 23.28 kg/m^2^) with the highest sensitivity and specificity (90.0%, 82.5%), clearly outperforming all other indices. This was followed by HC (AUC = 0.899; cutoff 100.0 cm), which showed balanced sensitivity and specificity (81.5% and 82.9%). Equivalent intermediate performance was seen for WC and WHtR (AUCs 0.861 and 0.858, respectively) with sensitivities of 77.6% and 81.3% and specificities of 82.0% and 80.6%. Neck circumference and waist-to-hip ratio were the weakest discriminators in women: NC showed a sensitivity of 72.2% and specificity of 71.4%, while WHR captured only 57.5% of obese women at the optimal cutoff with a specificity of 71.9%. The calculated Youden index identified the BMI cutoffs that provided the highest diagnostic accuracy for BF-defined obesity in our cohort, Figure 1C. The cutoffs were noted to differ by sex, at 26.32 kg/m^2^ in men and 23.28 kg/m^2^ in women, both below the conventional WHO obesity threshold, suggesting that lower BMI thresholds may be appropriate for identifying obesity in this Saudi cohort.

Across the six indices, BMI showed the most balanced combination of sensitivity and specificity for identifying BF-defined obesity in the overall cohort and in women, while WHtR achieved the best balance in men. WHR and NC produced the lowest Youden indices in all three analyses and are unlikely to offer additional discriminatory value beyond BMI and waist-based indices in this population.

## 4. Discussion

Obesity prevalence in Saudi Arabia is increasing, with obesity rates at 10% in the Western regions, and 14% in the Eastern region, and 4% reported in rural areas, with females being disproportionately affected by extreme obesity compared to males [24]. In the present study, we evaluated the discriminative performance of BMI, WC, HC, WHR, WHtR, and NC capacity in assessing obesity among Saudi adults. This approach enabled a focused assessment of how conventional, readily obtainable body-size measures relate to adiposity and how well they identify individuals with excess body fat. Among the anthropometric indices that were compared to the body composition index, BMI demonstrated the highest discriminative ability, followed by HC, WHtR, WC, NC, and WHR. The highest predictive performance demonstrated BMI as the best index, which reaffirmed its role as a robust obesity marker within the Saudi adult population. Nevertheless, it is increasingly recognized that in addition to total body mass, other anthropometric measures may capture distinct aspects of adiposity and cardiometabolic risk. Recent expert recommendations on clinical obesity have suggested that BMI should be supplemented by direct body-fat measurement such as BF% or supplementary anthropometry such as central anthropometric indices when true adiposity burden or obesity-related risk phenotype need to be determined [25].

Our results show that HC and WHtR have acceptable discriminatory ability and are thus useful in identifying individuals with obesity diagnoses. Previous studies have shown that WHtR had high diagnostic performance in screening for central obesity and was strongly associated with metabolic syndrome and cardiovascular risk factors in general, and even in Saudi adults [18]. This consistent evidence underscores the role of WHtR as a valuable, non-invasive measure of central fat distribution, a key determinant of cardiometabolic risk in this population. Our results also support those of another study involving a Saudi cohort, which revealed that WHtR and WC were superior to NC in identifying obesity, and the other indices, including WHR, demonstrated predictive ability for diagnosing obesity [17]. A systematic review and meta-analysis also found that WHR had stronger discriminatory power than BMI and waist circumference for several cardiometabolic risk factors in adults [26]. Our results support the use of sex-specific screening thresholds in routine clinical practice, particularly for waist-related measurements that are supported by findings from the SAUDI-DM study, which demonstrated the importance of using sex-specific cutoff values [27]. According to another study of the associations among several anthropometric measurements, including NC, WC, HC, BF% and BMI, and non-anthropometric components of metabolic syndrome in Saudi adults without diabetes, associations between multiple anthropometric indices and metabolic risk components were consistent for both sexes and exhibited no significant differences [28]. This finding is consistent with the results reported by Alzeidan et al., who showed that NC exhibited similar discriminatory power and strength of association with general obesity and metabolic syndrome across sexes [17]. The correlation analysis added another important layer of interpretation, which showed that strong correlations between BMI and BF% indicated that BMI broadly tracks adiposity in the study population. However, imperfect correlations would suggest that BMI and BF% are not interchangeable. Similarly, correlations between BF% and waist circumference, WHR, or WHtR would suggest that simple anthropometric measures capture part of the adiposity burden, particularly central body size.

The discriminative performance of these indices also differed significantly between sexes, which may be attributable to biological and contextual mechanisms in this cohort. WHtR was the leading discriminator in men, whereas BMI dominated in women, and WC and WHtR, which performed comparably to BMI in men, fell to a clearly lower level in women. This divergence suggests the sexual dimorphism of human adipose tissue wherein men tend toward an android phenotype, while women have a gynoid or pear-shaped phenotype [29,30]. We noted that in men, BMI, WHtR and WC performed comparably, with WHtR showing a small but significant advantage over WC, suggesting that any of the three may serve as a primary screening index for body fat-defined obesity in Saudi men. Hip circumference, neck circumference, and WHR demonstrated meaningfully lower AUCs and were significantly inferior to the leading three indices. The picture in women was distinctly different. BMI emerged as the most accurate discriminator outperforming all other anthropometric indices. Hip circumference ranked second, while WC and WHtR were lower and WHR was the weakest discriminator overall. These findings reflect the established sex differences in fat distribution between the sexes.

The strong and largely interchangeable performance of central indices in men can be related biologically, as men predominantly show an android (visceral) pattern of fat deposition characteristic of male adiposity, in which excess fat accumulates preferentially at the abdomen and is well captured by waist-based measurements relative to overall body weight or height [31]. On the other hand women accumulate adipose tissue both centrally and peripherally, with substantial gluteofemoral deposition that contributes meaningfully to total body fat but is not captured by waist-based indices alone [32]. Moreover, waist-based indices also do not capture peripheral fat depots and under-represent total adiposity in women, whereas BMI, which scales with overall body mass, integrates contributions from all depots and therefore tracks total body fat percentage more closely in this group [33,34]. Furthermore, life-stage and hormonal factors may contribute to the observed sex differences. Although the present cohort was relatively young, the gradual centralization of fat distribution across the female reproductive lifespan, accelerated by the menopausal transition, has been shown to shift the diagnostic performance of waist-based indices in older women [29]. In addition to these, shared environmental and behavioral exposures common to the Saudi population, including high prevalences of physical inactivity, energy-dense dietary patterns, and the resulting elevated burden of overweight and obesity [35,36], may modify the underlying biological differences in fat distribution between sexes. The improved performance of BMI in our population can be because it integrates contributions from all fat depots as total body mass and therefore tracks total adiposity more closely than central measurements. In the context of the present analysis, the strong ROC performance of WHR therefore supports the idea that height-adjusted central adiposity may be a more practical and informative index for identifying individuals with high BF% [26].

Our findings suggest that anthropometric indices used to approximate adiposity may benefit from sex-stratified interpretation. As discussed above, fat distribution differs systematically between men and women, with implications for how each index reflects total adiposity. A useful parallel can be drawn from the field of skeletal muscle assessment and sarcopenia where sex-specific reference standards are routinely applied because of physiological differences in body composition and tissue distribution, anthropometric indices may also require sex-stratified interpretation to improve obesity classification and cardiometabolic risk assessment. The revised European consensus on sarcopenia diagnosis (EWGSOP2) recommends different cutoffs for men and women for grip strength and appendicular skeletal muscle mass, on the grounds that muscle mass and strength differ inherently between the sexes [37]. A recent study showed a comparable principle in quantitative sensory physiology where sex has been shown to act as an independent biological modulator of perception even in healthy adults under standardized conditions [38]. Together, these studies suggest that sex-stratified interpretation may offer a more accurate framework than universal reference values, because of the physiological and biological influences on these indices. Clinical guidelines have already recommend different waist circumference thresholds for men and women when identifying abdominal obesity. Our findings appear consistent with this principle: the optimal cutoffs and the diagnostic performance of BMI and WHtR differed between Saudi men and women in our cohort, suggesting that sex-stratified interpretation of these indices may improve the accuracy of obesity classification in this population and could contribute to more accurate cardiometabolic risk prediction [39]. Confirmation in larger and more diverse samples would be needed before any change to current screening practice could be recommended.

This study had several notable strengths. First, ROC curve analysis enabled rigorous comparison of multiple anthropometric indices using standardized performance metrics, including AUC and CIs. Second, using the Youden index to identify optimal cutoff values yields clinically relevant thresholds directly applicable to the Saudi population, rather than relying on international reference values. Third, by including a wide range of anthropometric measures of general adiposity, central obesity, and body fat distribution, we were able to comprehensively evaluate both traditional and emerging indices. Finally, the focus on a Saudi cohort addresses an important gap in region-specific evidence and enhances the relevance of the findings for national screening programs and public health policies. This study also had several limitations. First, the cross-sectional design of our study with participants from the general population may limit causal inference. Second, our study population may not have fully represented all regions or demographic subgroups within Saudi Arabia; thus, the generalizability of the derived cutoff values may be limited.

## 5. Conclusions

In conclusion, obesity in Saudi adults can no longer be assessed through a single, universal lens. BMI remains a useful tool for identifying obesity as a standardized first-line measure, and BMI and BF% are better interpreted as markers of generalized adiposity, whereas waist circumference and waist-to-height ratio are more informative for metabolic risk stratification depending on who is being screened. Effective obesity screening in Saudi Arabia, therefore, requires moving beyond one-size-fits-all cutoffs toward sex-specific, population-relevant thresholds, a shift that is achievable using the same simple measurements already taken in routine care.

## Figures and Tables

**Table 1:** Participant characteristics (N = 1042).

Characteristic	Frequency	%
Sex		
Male	397	38.1
Female	645	61.9
Age		
18–30 years	597	57.3
31–50 years	345	33.1
51–60 years or older	100	9.6
Mean age (±standard deviation)	31.8 (±12)	
Marital status		
Married	374	35.9
Single	593	56.9
Divorced/widowed	75	7.2
Educational level		
High school or less	293	28.1
Diploma degree	63	6
University degree	539	51.7
Higher studies	147	14.1
Employment status		
Unemployed/unpaid housewife	227	21.8
Student	288	27.6
Private sector employee	140	13.4
Government sector employee	341	32.7
Business owner/retired	46	4.4
Household monthly income		
<5000 SAR	92	8.8
5000–10,000 SAR	239	22.9
>10,000–20,000 SAR	371	35.6
>20,000 SAR	340	32.6

Note: Data are frequencies and percentages unless otherwise specified. SAR, Saudi Arabian riyals.

**Table 2:** Participants’ anthropometric indices (N = 1042).

Anthropometric Indices	Data
Weight (kg)	72.5 ± 18.9
Height (cm)	164 ± 9
Body mass index (kg/m^2^)	26.8 ± 6.1
Body mass index level	
Underweight	135 (13%)
Normal	298 (28.6%)
Overweight	319 (30.6%)
Obese	290 (27.8%)
Waist circumference (cm)	84.93 ± 17.03
Hip circumference (cm)	104.81 ± 13.34
Waist–hip circumference ratio	0.83 ± 0.12
Waist-to–height ratio	0.517 ± 0.10
Neck circumference (cm)	35.04 ± 13.23
Body fat percentage	34.84 ± 10.53

Note: mean ± SD, n (%); body mass index was calculated as weight (kg) divided by height squared (m^2^) and classified according to WHO criteria.

**Table 3:** Pearson correlations between anthropometric obesity indices and body fat percentage, overall and by gender.

Index	Overall (N = 1042)		Males (N = 397)		Females (N = 645)	
	Pearson r	*p*-Value	Pearson r	*p*-Value	Pearson r	*p*-Value
BMI (kg/m^2^)	0.707	<0.01	0.837	<0.01	0.821	<0.01
Hip circumference (cm)	0.636	<0.01	0.766	<0.01	0.740	<0.01
Waist-to-height ratio	0.510	<0.01	0.828	<0.01	0.687	<0.01
Waist circumference (cm)	0.369	<0.01	0.789	<0.01	0.687	<0.01
Neck circumference (cm)	0.084	<0.05	0.531	<0.01	0.544	<0.01
Waist-to-hip ratio	0.075	<0.05	0.523	<0.01	0.304	<0.01

Pearson correlation coefficients (r) with significance levels are shown for each anthropometric index against body fat percentage (BF%).

**Table 4:** Discriminative performance of anthropometric indices for identifying body fat percentage–defined obesity in the overall cohort (N = 1042).

Index	AUC (95% CI)	*p*	Optimal Cutoff	Sensitivity (%)	Specificity (%)	Youden’s J
Body mass index (kg/m^2^)	0.924 (0.908–0.939)	<0.001	23.25	92.4	77.0	0.694
Waist circumference (cm)	0.824 (0.799–0.848)	<0.001	80.25	73.5	75.3	0.488
Hip circumference (cm)	0.887 (0.868–0.907)	<0.001	101.50	79.6	82.3	0.619
Waist-to-hip ratio	0.667 (0.634–0.700)	<0.001	0.88	46.1	84.6	0.306
Waist-to-height ratio	0.861 (0.840–0.883)	<0.001	0.48	79.2	79.2	0.584
Neck circumference (cm)	0.705 (0.673–0.737)	<0.001	33.50	67.5	61.5	0.290

AUC, area under the receiver operating characteristic curve; CI, confidence interval (computed using the Hanley–McNeil method); Reference standard: body fat percentage (BF%)–defined obesity (n obese = 686; n non-obese = 356). Optimal cutoffs determined by maximizing Youden’s J (=Sensitivity + Specificity − 1). The *p*-value tests the null hypothesis that AUC = 0.5.

**Table 5:** Sex-stratified discriminative performance of anthropometric indices for identifying body fat percentage-defined obesity.

Index	AUC (95% CI)	*p*	Optimal Cutoff	Sensitivity (%)	Specificity (%)	Youden’s J
*Males (N = 397; obese = 258, non-obese = 139)*						
Body mass index (kg/m^2^)	0.925 (0.900–0.950)	<0.001	26.32	78.7	89.2	0.679
Waist circumference (cm)	0.918 (0.891–0.944)	<0.001	92.00	83.7	85.6	0.693
Hip circumference (cm)	0.878 (0.845–0.911)	<0.001	103.00	80.2	82.0	0.622
Waist-to-hip ratio	0.806 (0.765–0.848)	<0.001	0.89	73.3	78.4	0.517
Waist-to-height ratio	0.932 (0.908–0.956)	<0.001	0.52	88.0	84.9	0.729
Neck circumference (cm)	0.819 (0.778–0.859)	<0.001	38.00	73.3	79.1	0.524
*Females (N = 645; obese = 428, non-obese = 217)*						
Body mass index (kg/m^2^)	0.930 (0.911–0.949)	<0.001	23.28	90.0	82.5	0.724
Waist circumference (cm)	0.861 (0.833–0.888)	<0.001	74.00	77.6	82.0	0.596
Hip circumference (cm)	0.899 (0.876–0.922)	<0.001	100.00	81.5	82.9	0.645
Waist-to-hip ratio	0.662 (0.620–0.705)	<0.001	0.76	57.5	71.9	0.294
Waist-to-height ratio	0.858 (0.831–0.886)	<0.001	0.45	81.3	80.6	0.620
Neck circumference (cm)	0.784 (0.750–0.819)	<0.001	32.00	72.2	71.4	0.436

AUC, area under the receiver operating characteristic curve; CI, confidence interval (computed using the Hanley–McNeil method). Reference standard: body fat percentage (BF%)-defined obesity. Optimal cutoffs determined by maximizing Youden’s J (= sensitivity + specificity − 1). The *p* value tests the null hypothesis; AUC = 0.5.

## Data Availability

The data presented in this study are available on request from the corresponding author due to privacy and confidentiality concerns.
